# The Hospital Nursing Supplement

**Published:** 1893-04-01

**Authors:** 


### "The Hospital" Nursing Mirror.

Being the Extra Nursing Supplement of "The Hospital" Newspaper.

[Contributions for this Supplement should be addressed to the Editor, The Hospital, 428, Strand, London, W.C., and should have the word "Nursing" plainly written in left-hand top corner of the envelope.]

### News from the Nursing World.

### A ROYAL GUEST.

When the Queen's eldest daughter, the Empress Frederick, visits a hospital in England, the administrative staff must feel they have to do with an unusually interested guest, as her practical knowledge of nurses and their work in Germany is vast and intimate. The Empress went over the Royal Hospital for Diseases of the Chest last week, and expressed herself much interested and pleased with all that she saw there.

### ROYAL PATRONESSES.

The Victoria Hospital for Sick Children at Chelsea is to have a cot endowed by the Children's Salon to the memory of the late Duke of Clarence. The Princess has chosen the inscription, "In Memoriam"—"The Albert Edward Victor Christian Cot." The hospital has a Royal patron, Princess Louise, who has for years past evinced deep interest in the patients and paid frequent visits to the wards. The Princess Victoria Mary of Teck has become patroness of the Victoria Home for Invalid Children at Margate, which was formerly the convalescent home in connection with the Children's Hospital at Chelsea. The beautiful new Home at Broadstairs has now replaced it in that capacity, and the old house known as "Churchfields," Margate, is therefore available for other service. It is utilised for some members of that vast army of invalid children for whom a sojourn in purer air than that of their poor homes is absolutely necessary. It is hoped that a stay at Margate will make healthy citizens of many delicate children, who would otherwise become permanent, instead of temporary, invalids. Princess Victoria of Teck is willing to receive donations for the Home at Churchfields, and has already collected £234 for it.

### GRATITUDE.

The gratitude and contentment of the patients at the East-End Mothers' Home were referred to by each speaker at the annual meeting, which took place at the Mansion House on the 24th ultimo. The Mothers evidently warmly appreciate the comforts which surround them at the home, as well as the atmosphere of kindliness pervading it. The outpatient department is also most valuable, enabling poor women to have the attendance of duly qualified nurses at their own homes. For this a small fee is charged, and has been proved to have an excellent effect in encouraging a provident spirit. When 3s. 6d. has to be saved in anticipation of the coming event, the forethought necessary, perchance, may embrace provision for other expenses equally requisite.

### VALUED WORKERS.

Most thorough appreciation of the nurses' work amongst the poor of Haggerston and Hoxton was exhibited by the speakers at the recent annual meeting of the Nurses' Association. Clergy and others testified to the great value of their skilful and unwearied exertions on behalf of the sick. The Bishop of Bedford and Mr. Pownall, chairman of the committee, appealed for increased support, and certainly it is well deserved by an association which accomplishes excellent and much needed work in these very poor districts.

### "A PLEA FOR SICK PAUPERS."

An important meeting at Chester the other day was presided over by the Duke of Westminster, who pleaded on behalf of the Northern Workhouse Nursing Association, of which the Duchess of Westminster is president. The Duke spoke in most encouraging terms of the improvement in those London infirmaries where trained matrons and nurses have been established, and hoped to see the example universally followed. A wish which all our readers will cordially endorse.

### FREE LIBRARIES.

Regular visitors to hospitals know well how heartily news from the outer world is welcomed by sick people. Current news, that is to say, such as is contained in yesterday's or this morning's papers, weekly illustrated journals, &c. Books, too, are always acceptable, though these should be wisely selected, as people in bed need to be supplied with cheerful literature in good type, if they are to get the maximum of pleasure at a minimum of effort. As yet patients in workhouse infirmaries are very insufficiently provided with books of an amusing kind. Granted that they cannot all read, still most of them appreciate illustraions. At one infirmary in the South of England a capital plan has been adopted by a lady visitor. Having begged from all her friends and acquaintances such stray volumes as they could spare, she has formed a nice little library. She goes at regular hours to both male and female wards, and gives out and changes the books. This is practical help, for it secures interest for the patients without adding work to the nurses, whose hands are generally quite sufficiently filled by their duties in large infirmary wards. Similar plans are in force at hospitals, and the monotonous lives of sick paupers surely require equal alleviations.

### ONLY FOUR MINUTES.

We hear occasionally—far too often, in fact—of a certain class of tragedy which takes place "during the temporary absence of the nurse." Patients, whose condition renders incessant care necessary, sometimes succeed in convincing their attendants that they can safely be trusted for short intervals. With the relaxed watchfulness, however, comes a catastrophe. This usually means a sudden or violent death for one unhappy creature, infinite distress for surviving friends, and professional ruin and life-long remorse for the nurse. At Birkdale, the other day, a lady was drowned, and the deplorable accident was a direct result of her "special nurse's" absence. At the inquest the latter explained having spent about four minutes in the public library whilst the poor lady remained seated outside it. The Superintendent of the Home gave evidence as to the care required by the patient, who was suffering from religious mania. This was corroborated by the brother of the deceased, who said that he paid for his sister to be constantly attended night and day by a special nurse. All this proves that the temporary absence of the latter was as inexcusable as it was unfortunate. Faithfulness in small matters is always proof of a conscientious nurse, but no details can be accounted small when mental disease is in question, and four minutes can be as fatal as four hours of neglect.

### LIBERALITY AT BURTON-ON-TRENT.

In the eighth annual report of the Burton-on-Trent Nursing Association special mention is made of the importance of systematic night nursing, and of the arrangements made by the institution to supply it. A relief fund provided beef-tea, milk, brandy, and wine to 77 patients, and other equal practical assistance is reported. The Home has been purchased, altered, and extended to give adequate accommodation for the district and private nursing staff, the handsome sum of £3,157 1s. being subscribed in the neighbourhood for the purpose.

### INGENIOUS DISINFECTION.

Householders, as well as nurses, have at times seriously to consider the importance of thorough disinfection—not the popular practice, which consists in placing stray saucers of Condy's fluid indiscriminately "diluted to taste," in corners of a sick room, nor the spraying of sweet perfumes, nor the sprinkling of toilet vinegars. These harmless customs have, let us hope, gradually given place to the stronger measures of reliable disinfectants of guaranteed strength. This progress largely results from the careful training received by nurses on these subjects, and by their subsequent faithful observance of the end aimed at. It is, perhaps, chiefly after an infectious illness has run its course that the householder's chief responsibility begins. The rooms have to be disinfected, and general experience shows this is one of the things needing intelligent supervision by the mistress or nurse. Otherwise holes in good carpets, or sulphur fumes issuing from imperfectly-closed windows or up the forgotten chimney may be looked for. The latest help which science has given in this important department of sanitation is described by Dr. Wynter Blyth, medical officer of health for Marylebone. The gas hitherto obtained by burning sulphur, and known as sulphurous acid gas, has been "liquified by cold and pressure in bulk, and can be bought in convenient hermetically-sealed tins, each tin being equal to about 2 lb. of sulphur." By cutting off a projecting lead tube the gas immediately hisses out. In fact this invention may be described as "disinfection made easy."

### FREE CONVEYANCE.

The District Nursing Association at Forfar seems to be doing good work. At the monthly meeting of the Executive Committee a variety of useful gifts were acknowledged. Mention was specially made of the practical help given to the nurse in her rounds by Mr. Greenhill and Mr. Petrie, who drive her without charge when she has to visit patients at a distance.

### BEREAVEMENT AND BITTERNESS.

The Committee appointed to inquire into the accusations made against a Scotch minister have now laid their report before the Presbytery of Inverness. Poor Mr. Simpson is exonerated from the serious charges of neglect brought against him by a brother of the minister who recently died from typhoid fever in his house. Mistakes and faults of management are shown in the evidence, as well as distress and agitation on the part of the Scotch minister and his wife. Probably they knew little of the nature of typhoid, and were inclined to exaggerate the danger of infection to their family. The two nurses had to combat drawbacks and difficulties, and to suffer personal inconvenience, but in one detail they certainly blundered. We read: "After the death, one of the nurses asked Mr. Simpson for a linen shirt. He brought out and offered some linen shirts of his own, but she said they were not suitable, as what she wanted was a night shirt." In consequence, the brother who had absented himself for a day or two on business, found on his return the dead man covered only by a sheet, and for this he blamed Mr. and Mrs. Simpson. But it was certainly no fault of the minister, and should not have been laid to his charge. Although one nurse said she thought that a shroud would arrive that night, the day shirt offered might have served the purpose, and a distressing detail of mismanagement would have been omitted. The two Scotch nurses had better acquire a little knowledge of modern customs, and then they will know that that ghastly invention "the shroud" is now frequently altogether superseded by the more familiar and ordinary garment.

### FAITHFUL SERVICE.

On the resignation of Nurse Morris, who has for two years served the Denbigh Jubilee Nursing Institution, the following testimony to her value was recorded: "The Committee regret having to report the intended resignation of Nurse Morris. During the two years she has been at work in the district she has performed her duties conscientiously, won the respect and love of those whom she ministered to, and has been welcomed and highly spoken of by all."

### ORDINARY NURSES.

The formation of a Nurses' Union in Paris gives hope for a general improvement in the prospects of our French sisters. At present nurses have to work from fifteen to sixteen hours daily. Private nurses are the victims of agencies which charge exorbitant commissions on their earnings, and the Union proposes to abolish this evil. We are told that ordinary nurses earn only 25 francs a month, and certificated ones get 28 francs; "for reasons of economy, however, the latter are rarely chosen." The "ordinary" nurse, therefore, holds no certificate, but surely she is not an altogether untrained woman?

### SHORT ITEMS.

A Nurses' Home has recently been opened for the accommodation of the staff of the Royal Hospital for Women and Children. The general condition of the hospital and the efficiency of the matron and her nurses was cordially acknowledged at the annual meeting.—The subject of village nursing has attracted the attention of the Earl of Winchelsea and Nottingham, who is warmly advocating the establishment of a duly qualified nurse in every English village. He thinks something might be done in conjunction with the National Agricultural Union.—The sum of £600 was raised at the bazaar lately held in aid of the Nurses' Institute at Northampton, which requires to be considerably enlarged to accommodate the number of nurses.—At the annual meeting of the Barry District Nursing Association, Dr. Treherne spoke of the desirability of adding one nurse to the staff to attend infectious cases, of which many occurred in the course of the year.

### THE DISABLED NURSE.

We have pleasure in acknowledging 2s. from Policy 2,414, and 2s. Policy 1,003, also 10s. from T. H. Neville.

### THE HOSPITAL ENDOWED BED.

We have received 2s. from M. A. Policy 1,033 this week.

### Management of Consumption.

(*Continued from page* cxciv., *Vol. XIII.*)

### VIII.—OCCUPATION FOR THE CONSUMPTIVE.

Part of the subject matter of this paper has already been treated of in a former article, but involving, as it does, the main elements in the management of consumptives, it will not be out of place to insist upon its importance once more.

All people are not in a position to choose their occupation; if they were, undoubtedly many would never have contracted this disease, since particular occupations play a great part in the production of consumption. In our crowded country, where the great majority of the working classes of our large towns depend upon labour in workshops at dusty employments for their daily bread, the mortality from consumption alone is appalling.

Broadly speaking, those employments which are dusty, and which must be followed within doors, are far more productive of consumption than those which are followed out of doors, that is to say, in the open air. Now, in the indoor employments two conditions are operating to produce consumption among the workmen: (1) the inhalation of dust; (2) the crowding together of many individuals in a confined space. The description of dust is a matter worthy of consideration, some kinds are much more injurious than others. That dust which is dry, and consists of minute hard, sharp particles, is the worst of all.

The constant inhalation of this dust has an irritating effect upon the lung, and if there is a predisposition to consumption, or if the workman inhaling the dust is weakly and run down in health, with an insufficient supply of fresh air and good food, the chances are great in favour of his taking the disease.

The statistics of mortality all show the baneful effect employments, in which there is a great quantity of dust, have upon the health of those employed. Among the most injurious occupations are those of needle grinders, stonemasons, cabinet makers, coal miners, cigar makers, and compositors. In some of these trades the average duration of life is not more than 30 years. When people engaged in these occupations die, it is possible, by suitable means, to see the minute particles of inhaled dust in the lung. It makes a great difference whether the dust of their occupation is in a dry form or wet. When wet it has little chance of flying about the workshop, and is less likely to be inhaled by the workpeople. A case in point will explain this. The grinders in steel used to grind and sharpen their wares with the grindstone quite dry, and this occupation was long regarded as the one attended with the largest mortality from consumption. After they ground with wet grindstones the mortality fell to an enormous extent. In London the cabinet makers and printers and cigar makers fall victims to consumption more frequently than those engaged in other occupations, chiefly on account of the fact that there is very little done in the way of metal grinding or stone work in the metropolis. By far the greater number of male consumptives applying at the East-end chest hospitals are either cabinet makers or compositors. We may dismiss, then, the subject of dust by saying that all dusty occupations are injurious to people with a tendency to consumption.

The second condition, crowding together individuals in a confined space, with an insufficient supply of fresh air, is just as important. This condition alone is quite sufficient to act as the main cause of consumption. Workrooms are always crowded; sometimes there is not sufficient ventilation, and the workpeople themselves object to have the windows open. The room becomes stuffy and hot; the greater part of the air breathed has been already expelled from the lungs, and every day spent in such an atmosphere is risking the onset of consumption. Suppose a case of consumption works in a room of this description full of people: the consumptive expectorates on the floor; this expectoration is quickly converted into dust, and it is the very likeliest thing in the world for every person in that room to inhale some of the spores of the tubercle bacilli, and there is no reason why those with a predisposition to the disease should not become consumptive. So all rooms badly ventilated and overcrowded, no matter to what trade or business they belong, must be looked upon as out of the question for people with consumption, and highly injurious for those who are as yet healthy, but still are predisposed to the disease.

Let the nurse then warn all consumptives she may have under her care to avoid if possible working at occupations in which there is much dust, not only for their own health, but also on account of the health of those whose duty it is to work beside them. Let it be explained to them why the windows of a workroom, all too insufficient for the number employed therein, should always be open. Advise consumptives to seek employment where they can be in the open air; and beseech the man, with a tendency to consumption, while there is yet time, to see the error of his ways, and if he can do so, even at a great personal and pecuniary sacrifice, let him give up his trade and take to some more congenial pursuit. How important fresh air is will be seen from the fact that cabinet makers, who work in shops, get consumption readily, whereas carpenters, who work in the open, are not very prone to the disease. It will of course often be impossible for the man with chronic consumption to give up that occupation upon which his daily bread depends. In that case he must be advised to have a spittoon by his side and to expectorate into it, instead of on the floor. He may also be persuaded to wear a respirator over his mouth if his occupation be dusty.

Without going into the exact nature of the different indoor occupations, it may be said that those in which chemicals, pitch, tar, and creosote are largely used are not injurious to consumptives, but, on the other hand, of benefit to them.

All this of course refers to the poor man, but the same general remarks apply to the man in better circumstances. All office work should be entirely given up by people with consumption, or a strong tendency that way, and an occupation at once light, free from worry, and entailing a large amount of outdoor exercise should be chosen instead.

### Novelties for Nurses.

It is always useful to know of materials which are serviceable as well as pretty, and certainly "unshrinkable flannels" rank foremost of these. Messrs. Barker and Moody, of Leeds, have some Ceylon flannels in most delicate shades, which will stand any amount of washing without losing their charming colours. Amongst other things, they make up into delightful summer dressing gowns, and they wear for years. The same firm also shows a large variety of white and cream flannels, which look very nice and are also "unshrinkable."

### Appointments.

[It is requested that successful candidates will send a copy of their applications and testimonials, with date of election, to The Editor, The Lodge, Porchester Square, W.]

Isle of Man.—Miss Meyer has been appointed Matron of Noble's Isle of Man Hospital. Miss Meyer was trained at the Bolton Infirmary and Dispensary, and was afterwards for eighteen months Charge Nurse at Noble's Hospital. She is, therefore, thoroughly acquainted with the institution, and has already proved a possessor of those qualities specially needed for a successful matron. We congratulate both Miss Meyer and the hospital which has secured her services.

### Nursing at Natal.

### A PRIVATE NURSE'S OPINION.

Members of the nursing profession in England are interested in hearing of the work of sister nurses in distant lands, and therefore will like to know something of the occasional periods of rest and relaxation.

Holiday amusements in some, at least, of the South African colonies vary widely from those enjoyed by nurses in England. As a nurse of many years' standing at home and in Natal, I have experienced both, and must admit that while, in the latter country, there is almost an entire absence of pleasant watering-places, public gardens, museums, and the like, still a holiday in Natal has a charm all its own. In a semi-tropical climate, the first desiderata for a pleasant holiday are plenty of shade, and an unlimited supply of fresh, open air, therefore the first natural instinct of the holiday-seeker is to reach the hills and valleys, which fortunately lie within easy access of towns and villages. We go by rail, if possible, and if not, by some other means of conveyance, not even despising the slow and ponderous, but time-honoured ox-waggon.

After one of the long periods of inaction and consequent low funds which are at present the frequent lot of colonial nurses, I was called in to a case of diphtheria in Maritzburg. The sufferer was a little boy about ten years of age, whose life there seemed at first but slight hope of saving. The attack was sharp, and the subsequent prostration severe and prolonged. After a week of great anxiety, however, he began slowly to amend, and I then found my store of firmness and patience taxed to the uttermost. He was not a very loveable child, being both selfish and obstinate, but, when I add that he was the only child of his mother, and "she was a widow." I need scarcely say that he was adored by her and sadly spoiled. It was, perhaps, this fact, joined to a certain quaintness of thought and speech, which made one feel sorry for the child in spite of his fits of sullen naughtiness.

I could fill a small book with the record of Eric's oddities, but it is rather of my pleasant holiday that I wish to write. When he was so far recovered as to take drives in a pony carriage, and was pronounced free from infection, his doctor recommended a change from Maritzburg, to thoroughly recruit his strength. His mother, as well as myself, had been in almost constant attendance in the sick room for nearly four weeks, and we were both feeling considerably out of tune. She, though not actually ill, had been greatly shaken by the severe strain upon her, and I had been suffering from a slight but obstinate form of diphtheretic sore throat. I was not sorry to hear it suggested that we all needed a change. Howick was the place fixed upon, and I was asked to be of the party. This village is situated about twenty-five miles up the line from Maritzburg, and is noted for its healthy climate and beautiful waterfall, 360 feet in depth. It is one of the favourite health resorts of the colony.

We drove in a cab to the station, then a small and rather primitive construction which has since been replaced by large and handsome buildings. On the platform Mr. Clarke pointed out two well-known citizens of Maritzburg, who were apparently travelling in the same direction as ourselves. One of these was Mr. Thornton, who has since risen to a distinguished position in the colony, and the other was Professor Cameron, an eminent traveller and scientist. It was well-known that these gentlemen, who held different views on political questions, had recently been engaged in rather violent disputes, and we noted with some amusement, the studied indifference with which they appeared to ignore each other's presence, and finally took their seats in different compartments of the train. This chance *rencontre* between the rival politicians afterwards provided us with some merriment.

My little patient was quite a well-known character at the station. Several of the officials smiled at him, and the good-natured guard who put us into our carriage said, "Good morning, Master Eric; glad to see you here again. You've been ill, eh?"

"Yes, that I have. But I pulled through this time," said the little fellow quaintly, with a solemn nod of his head. "I've had a narrow escape, though."

The country between Maritzburg and Howick is not so fine as some parts of Natal, but to us, on that bright morning, everything looked beautiful. I wonder if other people feel as I do when I get my first peep at the outer world after being confined for weeks within a sick room. How fresh and pure the air, how high the hills, how broad and open the green fields! "What a beautiful world!" has been at such times my first thought, and I have realized that the little circle which has been so engrossing, with its pressing duties and anxious cares, is, after all, but a very tiny part of the vast whole.

On this fair spring morning we sped swiftly past the grassy slopes, crossing now and again some tiny rivulet which trickled refreshingly over beds of fragrant fern, or through dense clumps of forest trees. We soon began to throw off the lassitude and depression which had long hung upon us. Even our little invalid was roused to interest, which brought a faint flush into his pale face and animation into his languid eyes, and, watching him, the careworn mother began to lay aside her fears, and to indulge in hope and confidence.

On reaching the picturesque station at Howick, bright with blooming flower beds and lovely tree ferns, we exchanged our seats in the railway train for the omnibus which there awaits the incoming trains. There are, indeed, two of these conveyances, belonging respectively to the principal hotels in the village, which lies about three miles distant from the railway station. Some rivalry exists, of course, between the drivers of these vehicles, and on the present occasion a great deal of friendly "chaff" was indulged in by our own Jehu, who had managed to secure all the passengers by the early train, and drove gaily on—while the less fortunate 'bus followed empty behind. Half an hour's drive brought us to the Castle Hotel, a very picturesque building, its double-storied frontage being built of grey stone, and turretted in imitation of a "castellated keep," but as our omnibus did not belong to this particular place, we were taken on a little further to the Howick Hotel, a large, low, and truly colonial building, with deep and cool verandahs surrounding it, on which opened many French windows, prettily draped with white curtains, which were like everything else most beautifully clean.

Our first sensation on entering this inviting place was hunger, for we had made but a poor apology for a meal before leaving home. We therefore ordered a second breakfast, which we heartily enjoyed, amongst most refreshing breezes, owing their coolness, no doubt, to the proximity of the waterfall. Having breakfasted and rested a short time, we decided that our first duty must be to visit the Falls, which are not more than five minutes walk from the hotel. On our way thither we passed several officers and men of the 11th Hussars, for though ordinarily a very quiet and sleepy little village, Howick at this time was rendered gay and busy by the presence of this regiment, which for some months was stationed there in consequence of a serious outbreak of fever at the camp in Maritzburg. Arriving at the drift, where numbers of Kafir women were busy washing clothes, their gay turbans or neckerchiefs giving colour to the scene, we took our seats upon some large stones near the edge of the chasm which Nature has here opened in the river-bed. A

few yards below the drift, where the stream seems still unconscious of the startling change awaiting it, a sheer drop of 360 feet causes one of the most beautiful waterfalls in Natal. This lovely cascade, sparkling in the sunshine, and throwing up clouds of snowy spray, was immediately opposite to our position, where, no doubt, thousands of people had sat before us through bygone years. This was proved by innumerable names carved roughly upon the stones around. Some had even been venturesome enough to leave their mark upon a rock jutting out from the very edge of the precipice.

*(To be continued.)*

### Attainable Perfection.

Private nurses really form quite an important feature of modern life, few households being without experience of their professional services. Whilst one family speaks gratefully of having had "a trained nurse, who was such a comfort to us all," another remarks, "We never want to go through such an experience again!"

Certainly the variety in employers is as great as that in nurses; and there is generally something to be said on both sides when the relations between the two classes becomes a strained one. A private nurse needs special as well as general preparation for her work. It is far too common a practice for the clever hospital nurse who gets run down by years of hard work to take a private engagement. She may do it voluntarily for a change or to get more money and longer holidays, or she may be forced into it by a matron who values her services, and yet knows that she is no longer fit for ward work without a break. "A year on the private staff" is therefore prescribed in a manner which leaves no option but to acquiesce.

Under the latter circumstances the nurse always goes to her first case unwillingly, and it is lucky if her principles are strong enough to prevent her patient from being made aware of her sentiments.

After her first experience is over a good nurse settles down, and shortly exhibits in the sick-room that skill which made her so invaluable in her ward.

It is a common characteristic of women that they seldom comprehend the need for preparing themselves for work of any kind.

Thousands of women say, and prove, that they are eager to work; nay, they are enthusiastic and indefatigable labourers. But if any one of them is told that she must pass through a course of training for the work to which she aspires, she quickly loses her interest, pleads inability to devote time and money to the preparation, and so gives up the scheme.

A few years back every middle-class woman who was obliged to earn her living (whether her taste or her education were commensurate did not matter) became a governess. It was imagined that any fairly refined young lady was quite competent to teach children.

We have learnt better now, and there are training colleges for teachers and deplomas of qualifications to be obtained before this most important avocation can be successfully embraced.

The need for instructing our nurses has also been thoroughly realised, and we see that the partially trained attendant has but little chance of obtaining a good appointment now-a-days.

The careful preparation secured for district nurses who aspire to be Queen's nurses is excellent, and we only wish that those of our sisters who take up private work were equally well qualified for it.

The greater freedom enjoyed by women "working on their own account" is the attraction and also the bane of these nurses. Day after day, and week after week, they are left to their own discretion.

The doctor's daily visit over, no one comes near them who knows more, or even as much as they do, of the elements of skilled nursing. It is a great temptation to many to make capital of this fact, for they are aware that their most foolish restrictions or indulgencies will not be gainsaid.

The intelligent refined mistress of the house may have her feelings outraged each time she enters her sick daughter's room by the "bad style" of the nurse's get up, or her inattention to details which, though perhaps not strictly nursing matters, are still included in a woman's province. A lady often will not speak of the neglected duties because she is fearful of offending the attendant on whom her daughter's mental as well as physical comfort depends. In this she is wise, for the woman whose "style" is bad is oftentimes too vain to bear correction meekly, however gently it be given.

Sometimes, however, imperfect private nursing is entirely due to ignorance, as in the case of the good worker from a hospital who has never been instructed in a hundred details needful for finished attendance in a sick room.

It is the misfortune, not the fault, of the ward nurse that she has been cut adrift from her familiar moorings and left to steer her course alone in unaccustomed scenes.

Training for private nursing is absolutely necessary to ensure perfection. The experience should be obtained by systematic teaching, either in private wards or good nursing homes. It would not take long for an intelligent trained nurse to acquire the requisite details, but they should be properly laid before her and shown to her practically. She should be a pupil only and not put to work alone. Certainly, such knowledge should never be bought at the expense of a patient or his household, which is the plain state of things when anyone goes straight to a sick room without any preliminary preparation for private nursing. In a ward a charge nurse's position is clearly defined, her authority, if wisely exercised, is unquestioned, and her supervision covers a large area. Therefore her transition to a private room is a great and a puzzling change. Her field for management is concentrated; she often has to sit motionless when incessant movement would be more congenial to her, and she must conciliate the household, which is suspicious of her innovations. She has to humour the whims of the fanciful patient who knows nothing of those serious trials of poverty and suffering so familiar to herself, and she must not be impatient of the trifles which are well nigh insupportable to those unaccustomed to patient endurance of discomfort. Sympathy is the keynote to success in all kinds of nursing, but it is harder to exercise this quality towards those in comfortable outward circumstances than it is to pity the sick, on whom the additional burden of poverty is laid. However, no woman intending to achieve perfection in private nursing should rest content till she has attained universal sympathy with suffering of every degree.

### Notes and Queries.

### Queries.

(90) *Servants.*—Can you tell me if there is a pension fund for servants?—*Policy* 2,414.

(91) *Nursing.*—Will you recommend me a good book on fever nursing?—*Hereford.*

(92) *Switzerland.*—Will anyone advise me as to an institution for nurses in Switzerland?—*Nurse A.*

(93) *Training.*—Information as to the training in France or Italy wanted by an English girl.—*Sister Jessie.*

(94) *Medical Women.*—Information as to examinations after private study, &c., wanted.—*Violet.*

(95) *Surgical Nurse.*—Having seen advertisement in The Hospital for a surgical nurse, I should be greatly obliged if you would send particulars, &c.?—*L. J. N.*

### Answers.

(90) *Servants (Policy* 2,414).—Not that we know of.

(91) *Nursing (Hereford).*—"Notes on Fever Nursing," by T. W. Allen: publisher Lewis, 136, Gower Street, London; price 1s., or any good Nursing Book.

(92) *Switzerland (Nurse A.).*—You would obtain information on this subject at the Bureau at Cannes. Get a notice put up in the public library there. You might advertise in a Cannes newspaper stating your wishes. Be sure and make careful inquiries before joining any association whatever.

(93) *Training (Sister Jessie).*—Read "Howe to Become a Nurse," by Honnor Morten, published by Scientific Press, 428, Strand, and write direct to the superintendents of the hospital, given.

(94) *Medical Women (Violet).*—You had better write to Secretary. School of Medicine for Women, Handel Street, London, W.C., for all the particulars.

(95) *Surgical Nurse (L. J. N.).*—Your undated letter gives us no clue as to which of our numerous advertisements you refer to. You specify neither the date of the paper nor the page on which the advertisement appears.

### Thoughts About Pictures for Holy Week.

Among the various sacred subjects which attracted the Italian painters, the "Last Supper" is one of the most interesting in which to trace the variety of their conception and treatment. The painter who first attempted a representation of this scene was Giotto, and his fresco is still preserved in the refectory of Santa Croce at Florence. Giotto stands but second in point of time on the great roll of Italian painters who added to each other's heritage till they filled the world with their renown, but appreciation of the more perfected methods of the later artists need not destroy the charm which Giotto's earnestness and simplicity lend to his work. He is earnest, for he has always a meaning worth taking some trouble to make out. He is simple, for he concerns himself not at all with details outside his purpose. He represents the "Last Supper" as taking place in a lofty room, resembling a monastic refectory of his own day. The Apostles are seated on one side of a long table, six on the right of our Lord and five on the left. Judas on the opposite side of the table, without a halo, and with his hand in the dish, turns half-round with a sly, bad expression. The table is covered with a white cloth, and bears a few simple dishes, among which a plate of olives may be distinguished, and flagons half full of red wine. This simple framework, which has the merit of showing the faces and gestures of our Lord and all the Apostles unimpeded, was adopted by Giotto's successors, and became the conventional method of representing the scene. Standing before the fresco, which takes up the lower end of the bare disused wall, you examine the faces of the Apostles, and try to enter into the aim of the painter. It is not easy, for it requires little knowledge of painting to see that most of the faces have been restored out of all likeness to their original colouring. The St. John, leaning across the Saviour, with his head bent low, conveys nothing to you. That old man, too, on the right, with his face half hidden in white hair and beard—you are sure Giotto never painted that unmeaning face for St. Peter. But see that eager young man near the end, probably St. Jude; he has risen from his seat in agitation, and bends forward towards the Lord. You may see faces like that sometimes now in Italy among the younger Jesuits, full of zeal, full of devotion, and strivings after purity; but Giotto's face tells more—it asks a question; and as you gaze you seem to hear the humble, awe-struck words "Is it I?" The words are like a clue to the whole composition. Look along and you see the question repeated in wonderful variety of character among the long line. Each is putting it to himself or to his neighbour or to his Lord, and you somehow feel that the purest hearts are the most deeply stirred.

They gaze on one another with strange fear,
What! did the Master say one present here
Should yet betray Him? Is there round this board
One of our number faithless to the Lord?

Who may it be? say, Master, is it I?
What wild heart-throbbings wait on the reply;
The words pass round with mingled pain and dread,
Till the sad answer cometh, "Thou hast said."

"Lord is it I?" I too have dared to come
And eat with Thee as in that upper room,
Thy close familiar intercourse to claim,
And breath,e as one beloved, Thy sacred uame.

And now I turn to ask Thee, "Is it I
Who shall betray Thee?" Waiting the reply
As anxiously as those fond hearts who knew
Love might be false ere Thy words prove untrue.

"Lord, is it I?" Am I in heart Thine own,
Not for vain use or fashion's sake alone,
Not even for the hope Thyself doth give,
But simply Thine because in Thee I live?

"Lord is it I?" And have I lingered near,
Thy works to mark, Thy wondrous words to hear;
Been counted by the world around Thy friend,
But to be basely faithless in the end?

"Lord, is it I?" Would I for Thy dear name
Bear the world's hatred, scorn, contempt, and blame?
Or would I draw myself unharmed away,
And with a kiss my blessed Lord betray?

"Lord is it I?" It is not yet too late,
Let me repent while I the answer wait;
Let bitter tears of penitence be shed,
Lest Thou to *me* shalt answer "*Thou hast said.*"

E. M. L.

Turn away from the noble simplicity of this little company of men, the working of whose minds is reflected with the vividness of childhood on their faces.

Another refectory; this time you are in the convent of San Marco, and a glowing mass of colour and a far more elaborately painted composition than Giotto's is before you. It is the work of Ghirlandajo. "How beautiful," is the first exclamation; "What lovely trellis work of leaves and flowers hanging from above; what noble architecture, and the cat in the foreground, how perfectly natural! See the embroidered table-cloth and the fruit and cakes on the table, how wonderfully real. The colouring of those robes is magnificent, and the figures so well grouped. Ah! I see it is meant for the Last Supper." Yes, that is remembered at last, but when remembered it, makes no difference in your admiration. You examine each face, and in each you find a suitably reverent and subdued expression. Some of the figures raise their hands in awe, others look much surprised, but all give the impression of having been grouped for effect, of having assumed the right attitude because directed by the painter. Nothing is spontaneous, nothing real, except the unimportant accessories. But this decorative part is unquestionably beautiful.

Again another refectory—that of St. Onofrio—and now we are sitting before the work of Raphael. It is Raphael in his young days, before he had left his master Perugino's hand, and so little like his later style that only quite lately has it been proved to be his from the almost invisible signature running round the hem of St. Simon's garment. You will not understand this fresco without silence and time. You are disappointed at first, you expected something striking and unmistakably powerful, and are surprised to find the same long refectory table and the same grouping of the Apostles, with Judas in front. But look long and thoughtfully; try to exclude everything from your mind but that quiet upper chamber; enter into its atmosphere and let the stillness, the mournful silence sink into your soul. Then remember that these men are gathered round their Master for the last time; that separation, shame, and sorrow, await them on their departure from the room, dimly understood by them from the Lord's own words, fully foreseen by Him down to the last pang of death. Then you will see that the painter has realized for us the very aching desolation of the disciples' hearts, relieved yet intensified by the Master's presence. "But now I go my way to Him that sent Me; and none of you asketh Me whither goest Thou? But because I have said these things unto you, sorrow hath filled your heart." The *Last* Supper is before us here; the moment's pause before the breaking of the storm which is gathering in their sight.

We turn to the conception of Andrea del Sarto with all the more eagerness, that by the nature of the subject the lovely Madonna face of his wife, which always pleases, yet palls a little by its perpetual presence, is excluded. Here the interest centres markedly round the figures of our Lord, with St. John reclining on His breast, and that of St. Peter. The St. John is exquisitely pure and beautiful. The face is that of an archangel, unshadowed by consciousness of wilful sin, wholly absorbed in entire devotion to His Master, soothed and raised to a higher atmosphere by the absolute confidence in His love, expressed by the clasped hands. In St. Peter's face can be read all his past history of failure and enthusiasm, and there is not wanting a presageful touch of the coming fall. The moment chosen seems to be the one immediately preceding our Lord's warning.

Lord, I am thine ; 'twas Thy unerring hand
Which beckoned me to follow, yet long years
Have stamped me with the world's seal ; and no tears,
No agony of will can make me stand
Firm 'mid the waves. Eldest of all Thy band
No young, unstained and virgin heart is mine,
Like his who leans with fingers closed in Thine,
His head upon Thy breast. Yet I had planned
To shield Thy life from danger with my own,
Turn hardship from Thee, smooth Thy path of pain,
And win by service, love from Thee, and grace.
Now in Thy darkest hour will I atone
For shameful failure—never to fail again.
Why lookest Thou on me with that pitying Face?

The great painting of Leonardo da Vinci, of which the fragments are still to be seen on the wall of the suppressed monastery of Santa Maria delle Grazie, near Milan, is too familiar to everyone in copies and photographs to need description. It is, perhaps, only in presence of the original, mutilated though it is, that its full dramatic force is felt, and the grandeur of the whole composition understood. But the beauty and dramatic power of the composition represents only half its value. It is Leonardo alone who has been able in treating this subject to make the Christ the central point of influence, the one absorbing Personality, round Whom the conflicting emotions of the Apostles stir and are stilled. The face has been repainted, but, happily, can still be studied at its best in the gallery of the Brera close by, where the original study for the larger composition is preserved. The attention strays back again and again from the subtle touches of character and passion shown in the portraits of the Apostles to the kingly figure of their Master. The bowed head, the downcast eyes, the whole attitude, with the motion of the arms and hands, seem to repeat with divine resignation, which His silence confirms, the fateful words, "One of you shall betray me." Perhaps no moment of bitterer humiliation could have been chosen than that in which the Saviour showed His knowledge of the treachery of one, of the faithlessness of all His friends, and it is because Leonardo has not shrunk from the intensity of the humiliation, and yet has conceived the sufferer as King and as God, that this figure is for many the representation most approximate of all in sacred art to their ideal of the Man Christ Jesus.

### Everybody's Opinion.

*[Correspondence on all subjects is invited, but we cannot in any way be responsible for the opinions expressed by our correspondents. No communications can be entertained if the name and address of the correspondent is not given, or unless one side of the paper only be written on.]*

### CONVALESCENT HOME.

"E. M. S." writes : I should like to draw the attention of The Hospital readers to a Home or boarding house, presided over by two trained nurses, at Oakdene, London Road, St. Leonards-on-Sea. I have spent some weeks there, and cannot speak too highly of the comfortable arrangements.

### BORROWED WITHOUT ACKNOWLEDGMENT.

"A reader" writes : I have been perusing the American paper, "The Trained Nurse," and under the title of "The Editor's Table" I was amused to find a rather strange medley. There are eight or nine lines of introduction before the tenth begins "Wiser heads than ours." The words seemed familiar, and I read on and found an almost literal reproduction of an interesting article which appeared in The Hospital of September 3rd, 1892. "The Trained Nurse," however, omits to mention from whence it has culled this contribution, and the introduction tends to give a false impression as to the authorship.

### NURSES FOR NORFOLK.

With reference to the note published under this heading last week, we have received the following letter. It will be seen that it does not touch the point at issue, seeing that no responsible Committee is justified in entering into a speculation of this kind at the expense of the nurses or other trained women workers :—

Referring to a paragraph in your issue of this date, allow me to say that the salary of £25 for a nurse is fixed not with a view to appropriate an undue portion of her earnings, but for the purpose of limiting expense in case the experiment should prove unsuccessful. I may add that two nurses have offered their services, and that the undertaking starts with a fair prospect of success.

J. J. Coulton, Chairman of Weekly Board.

West Norfolk and Lynn Hospital, Lynn,
March 25th, 1893.

### For Reading to the Sick.

### CHRIST'S LOVED ONES.

We may use the prayer, "Lord, he whom Thou lovest is sick," with the same confidence now as did the sisters of Bethany when they besought their beloved Friend and Master to come and heal their brother. For Christ loves you, He loves me, He loves every one of us as tenderly as when He trod the earth, and will listen with the same compassion to our cry for help. And yet we turn from His powerful aid because we cannot see Him, nor even guess that we have His presence with us through all our weary hours of pain. Do we ever ask ourselves who it was that calmed our troubled hearts and soothed our aching brows for a little while, and made us brave to face the coming agony? It was Christ watching us from His heavenly home, gently making "all our bed in our sickness," luring us to rest in Him when all other rest is as weariness both to soul and body ; He bestows on us the ineffable calm of a will in union with His will, the peace which passeth all understanding, and at last "He giveth His beloved sleep." "He whom Thou lovest." This is the blessed title which all the children of God may claim, "the happy title," says a writer, "which we may humbly share with Lazarus and St. John, those two whom Christ loved with a special sympathy on earth ; blessed beyond measure is the thought in the time of sickness and of suffering, that thus we may be spoken of in heaven." But instead of turning to this loving Lord, we go on, full of weariness, tired of our lives, tired of ourselves. How willingly would we throw the burden of existence down to be rid of this gnawing pain, this diseased body ! At least, so we say, and perhaps, in a measure, so we think ; but life is very sweet, and in moments of ease we cling to the pleasant ties of wife, and child, and kin. They are the purest, most wholesome instincts of our nature, yet even they fail us, unless behind them stand the Heavenly Father and the "Friend who sticketh closer than a brother." These alone are our true hope and consolation, and Christ will come to the aid of His loved ones, though He may seem to tarry. He whispers in our ears, "Fear not, for I am with you ; I will strengthen you, I will help you." If we are His loved ones, do we also return that love? Alas! how cold and dead we often feel, yet He looks upon the faintest spark of desire ; He fans the smouldering embers with the breath of His holy spirit till our hearts glow again in response to Him, and we can plead our own cause and say, "Lord, he who loves Thee is sick, come and heal him body and soul."

### An Infallible Cough Mixture.

A House Surgeon's Story.

(*Continued from page* cxcviii., *Vol. XIII.*)

II.

It was Sunday. I had finished my work in the wards, and was looking forward to dinner and a quiet afternoon, with perhaps a novel and my pipe as a preliminary to a couple of hours of oblivion in sleep, when the housemaid came to tell me that a woman desired to see me immediately in the waiting-room. I found a woman with a shawl over her head and one of the younger Baileys. They rose from the seat as soon as I entered, and with glistening eyes and anxious looks asked me to come with all speed to Bailey's house. A "dreadful accident had happened to the baby, and it was as much as they were worth if it was alive now."

I was under no obligation to obey this call, but the claim of common humanity urged me to attend to it, whilst the thought that in the case of an inquest my conduct would be placed before the public in no flattering light if I stood upon my rights and refused to go, also influenced my decision.

In less than a quarter of an hour I was pushing my way through a nondescript crowd of chattering women and children, in every stage of cleanliness and *dishabille*. The news that the last addition to the Bailey family had been shut up in the turn-up bed, and was believed to be smothered had spread with characteristic rapidity. Housewives who ought to have found the preparation of Sunday dinner a sufficient reason to stay at home, fled from their hearths to learn the real news and share in the excitement; whilst the younger members of the community caught the contagion of their elders' example, and with them came swarming around the door which opened upon the scene of the calamity. I pressed forward, the crowd giving way before me as soon as I was recognised, the babel of voices sinking lower, and the dismal lamentations over the fate of the unhappy child being for the moment stopped by my appearance.

Within the house the crowd seemed more dense than on the flags, and it was with difficulty that I edged my way to the side of the sufferer, who was on the lap of a neighbour, resisting with many vigorous cries the united attempts of three well-meaning matrons to force some brandy down his throat. The mother was rocking herself to and fro upon a chair in a corner, heedless of the kind attentions of a group of would-be comforters, who were bidding her with the greatest fervency to "bear up, and look on the bright side of things." Bailey himself had taken a back place, and was sententiously watching the course of events, ready to respond to any call made upon him—he was apparently well used to the domination of female outsiders—whilst the old Bailey and the rest of the family were standing or sitting about, wherever they could find an inch of space, their staring eyes and blank faces telling that they were prepared for the worst.

My first action was to clear the room. If the baby had been partly suffocated it would need the fresh air that this score of people was taking from it, and it was impossible to attempt any treatment hemmed in by such a crowd. Having accomplished this with difficulty, and with some show of unwillingness on the part of those I expelled, I turned my attention to the child. The little one was more frightened than hurt, and must have suffered more at the hands of its Good Samaritan friends than by its temporary enclosure in the turn-up bed. How it got there none of the family knew. It was suddenly discovered to be missing. The time of its disappearance could not be fixed; among so many, one more or less either way was not of much account.

When the mother heard that her child was saved she flung herself on her knees before it and literally fell on its neck and kissed it. Then she took it in her arms, sat down, and after the first frenzy of her joy had subsided, gave me an incoherent history of the mishap.

"I'd washed the other childer, and then I washed the baby and gave him to Sarah Ann to hold, till I got the dinner ready. Then he fell asleep, and she put him on the bed, and pared the potatoes for me. When I had the potatoes on, and the puddings boiling—we always have suet dumpling on a Sunday—I went upstairs to make the beds, and while I was up, Bailey came in, and seeing the bed all down, straight way goes and shuts it up. He didn't notice the child, it was so covered up with blankets and shawls. And that's all I know till they come and tell me that the darling was smothered to death in the bed."

And she fell again to smothering the little morsel with her kisses, as if it had not run sufficient risk of suffocation already.

As soon as the other children saw that their little brother was out of danger they gathered more courage and came from their places of retirement near the wall, drawing near to what was evidently the centre of attraction, the round deal topped table, already spread as for a meal when the baby's peril put a stop to the preparations. A girl of fourteen, a handy useful child, began to busy herself about the pans on the fire, and after prodding inside one of them with a fork, she announced to the evident pleasure of her waiting brothers and sisters, that the dumplings were ready. There was a scurry for places; some crowded to the table, others seated themselves on the boxes and short logs of wood at one side of the hearth, and the mouths of all were watering in anticipation of the coming feast. The father lifted the big pan from the fire, and the maid with the fork began to unearth the steaming dumplings from its interior, when suddenly, as if something had been overlooked, one of the younger lads shouted, "And the sarse."

In an instant two of his brothers darted to the cupboard in the corner, and came back in triumph, not with a bottle of some much-advertised condimental mixture, but with a bottle of that particular cough medicine to which old Bailey was so partial. With a look as if he had done a praiseworthy act, the lad put it in the place of honour in the centre of the table.

I had watched all this whilst giving some last instructions to the mother about the baby, and I saw the father's brow grow angry and heavy as the cough mixture appeared. It was too late, however, to avert the mischief, and his face blushed as he tried to explain the circumstance.

"The children like it," he apologised, "and we didn't think it was doing them harm. If it was good for them when they were ill, we didn't think it could do them any harm when they was well, and they liked it so well that we didn't like to refuse them."

Here, then, was the solution of the mystery of old Bailey's fondness for his cough mixture, it was not only a cough mixture, it was pudding sauce for all the family. It is needless to say that the old man received no more of the palatable, soothing mixture, and as I slowly walked back to my own dinner I could only wonder once again at the wondrous mixture of humour, pathos, and tragedy called human character. I afterwards learnt that every Sunday the Bailey family had dined on dumplings, to which the famous cough mixture was the unfailing accompaniment.

### Wants and Workers.

Can anyone tell me of an institution which would receive an epileptic girl of twelve? A donation of £10 is offered, but nothing more can be given towards her support.

Will any reader holding the L.O.S. diploma give a few hints as to the examination to help a fellow nurse who is trying for it unaided? Address Nurse B., 78, Gower Street, London.

### The Book World for Women and Nurses.

[We invite Correspondence, Criticism, Enquiries, and Notes on Books likely to interest Women and Nurses. Address, Editor, THE HOSPITAL (Nurses' Book World), 428, Strand, W.C.]

UNSEEN FOUNDATIONS OF SOCIETY. By the DUKE OF ARGYLL. (John Murray.)

This substantial volume dealing with some of the most pressing questions of the day will be cordially welcomed by the large proportion of readers for whom the Duke of Argyll is "a name to conjure by." The book is "an examination of the fallacies and failures of Economic Science, due to neglected elements." It is a plea for sounder methods and truer principles in a science which has become in many minds a bye-word and reproach. It is to a great extent a review of the earlier systems of Political Economy, weighing them in the balance and showing to what extent they have been proved in the history of the world to have been found wanting. But the chapters which will excite most attention and best repay a careful study are those dealing with what, for want of a better word, we must still continue to call Capital. The Duke of Argyll would substitute the word "conceiver" for capitalist, defining the functions of capital as "the functions of mental labour wielding the resources which antecedent labour of the same kind has placed at its disposal." In his just deprecation of the fallacies by which the wage-earning classes are led to regard themselves as almost the sole factors in the production of wealth, the author does not appear to assign a sufficient place of importance to the skilled artizan. It may be claimed with safety that not all the mental power is on the side of the capitalist, though in the starting of new enterprises, from which the illustrations are chiefly drawn, the projector's genius will certainly be the most conspicuous element. In nine cases out of ten it must, however, be recognized that the skill, foresight,, and inventive faculty are all on the side of the wage-earner, while the function of the capitalist is confined to receiving the profits. In dealing with the question of population, the author considers we should learn confidence in the future from the lessons of the past. "It may well be amusing to us now, but it is also highly instructive to find that the growth of London even in the days of Queen Elizabeth was attracting such attention, and exciting such alarm that she directed an edict against it, in the form of a direct prohibition to build any more houses. Most of the evils she dreaded and enumerated when London contained probably little more than a hundred thousand people are very much the same as those which now affect our own imaginations when its population has swollen to the enormous amount of upwards of 4,000,000. They were the danger of epidemics; the difficulty of governing well such multitudes; the difficulty of supplying them with food; the multiplying of the very poor; the increase of manual labourers beyond the means of employment; and the impoverishment of the country and of other towns for lack of inhabitants. The population of London has been multiplied by more than 40,000 times since 1580. It is by far the largest city in the world, yet it is a city more free from epidemic disease than any other, and on the whole much healthier. Above all other wonders about it, day by day its four millions of mouths are fed as it were automatically without a thought or care on the part of its municipality, simply by the working of the self-interest of innumerable individual minds." The author concludes with an earnest protest against over-legislation, and enforces with the familiar and convincing eloquence of his earlier works "the grand conclusion, reached by the purest and strictest logical process, that the real welfare of everybody is bound up with the real welfare of everybody else."

Literary Blunders. By H. B. Wheatly, F.S.A. (Elliot Stock.)

There is always something pleasing in the contemplation of other people's blunders. Even when the blunder disclosed is one which we have neither the wit to perpetrate nor the knowledge to find out, it tickles the vanity in obedience to some mysterious law; but the charm is no doubt enhanced when we stand on the proper level of superiority. Mr. Wheatly has collected a very happy set of Literary Blunders in the new volume of the "Book-lover's Library," and no book can be more cordially recommended for taking up in a depressed half-hour to restore some harassed blunderer to a renewed enjoyment of the absurdity of things in general. Under the head of "Bulls" we get the following passage from the report of an Irish Benevolent Society: "Notwithstanding the large amount paid for medicine and medical attendance, very few deaths occurred during the year." A country editor's correspondent wrote: "Will you please to insert this obituary notice? I make bold to ask it because I know the deceased had a great many friends who will be glad to hear of his death." It is something of a shock to catch Mr. Gladstone tripping in Bible history, but most Sunday-school children could set him right when he says "The fierce light that beats upon a throne is something like the heat of that furnace in which only Daniel could walk unscathed." Some of the blunders of translators recorded are very neat, especially those from English into French. It seems that "Walker, London" figures in the "Bibliothèque Universelle" under the title of "Londres, qui se promene." The old farce, "Hit or Miss" turns into "Frappé ou Mademoiselle," while the adjective "woe-begone" is rendered in all good faith "ainsi douleur, va-t-en." Some of the school-boy blunders sound almost too good to be genuine. What does the profession say to this new organ of the body? "He had chronic disease—something the matter with the *chrone.*" But a bolder shot was made by the boy who asserted that "John Bright is noted for an incurable disease." There is a fine confusion of ideas in the brief biography, "Esau was a man who wrote fables, and who sold the copyright to a publisher for a bottle of potash." The following answers were supplied by Professor Oliver Lodge from papers written by candidates under examination in the Science and Art Department: Question 12, "State what are the conditions favourable for the formation of dew." Answer, "A body of gas as it ascends, expands, cools, and deposits moisture; so if you walk up a hill the body of gas inside you expands, gives its heat to you, and deposits its moisture in the form of dew or common sweat. Hence, these are the favourable conditions; and, moreover, it explains why you get warm by ascending a hill, in opposition to the well-known law of the conservation of energy." Question 11, "Explain why, in order to cook food by boiling, at the top of a high mountain, you must employ a different method from that used on the sea level." Answer, "It is easy to cook food at the sea level by boiling it, but once you get above the sea level, the only plan is to fry it in its own fat. It is, in fact, impossible to boil water above the sea level by any amount of heat. A different method, therefore, would have to be employed to boil food at the top of a high mountain; but what that method is has not yet been discovered. The future may reveal it to a daring experimentalist."

